# Watchful Waiting for Children With Acute Otitis Media: Frequency of Use and Outcomes in Clinical Practice

**DOI:** 10.1093/jpids/piaf104

**Published:** 2025-11-12

**Authors:** Timothy C Jenkins, Adam L Hersh, Amy B Stein, Rashmi Narayan, Alice Eggleston, Holly M Frost, for the DISAPEAR Study Group, Andersen Leisha, Gilbert Aiden, Hasan Aylin, Jensen Hannah, Keith Amy, Milanes Mirta, Morin Theresa, O’Leary Sonja, Nelson Barbora, Patel Payal, Rinehart Deborah, M Seibert Allan, Stanfield Valoree, Willis Park

**Affiliations:** Division of Infectious Diseases, Department of Medicine, Denver Health and Hospital Authority, Denver, CO, United States; Division of Infectious Diseases, Department of Medicine, University of Colorado Anschutz Medical Campus, Aurora, CO United States; Division of Infectious Diseases, Department of Pediatrics, University of Utah, Salt Lake City, UT, United States; Center for Health Systems Research, Denver Health and Hospital Authority, Denver, CO, United States; AllianceChicago, Chicago, IL, United States; AllianceChicago, Chicago, IL, United States; Division of Infectious Diseases, Department of Pediatrics, University of Utah, Salt Lake City, UT, United States; Office of Research, Intermountain Health, Murray, UT, United States

**Keywords:** acute otitis media, watchful waiting, antibiotic stewardship, pediatrics

## Abstract

Among 140 579 pediatric acute otitis media (AOM) visits, watchful waiting was used in 15.6% of cases and was associated with similarly low rates of treatment failure and adverse events as immediate antibiotics. Scale up of watchful waiting may be an important approach to reduce unnecessary antibiotic exposure for AOM.

## BACKGROUND

Each year, about 10 million antibiotic courses are prescribed to children for acute otitis media (AOM), though over 80% of cases resolve without antibiotics.^1-3^ Despite limited benefits, immediate antibiotics (an antibiotic to take right away) are prescribed in over 80% of cases, putting children at unnecessary risk for antibiotic-related adverse events.^4^^,^^5^

Watchful waiting is one approach to reduce unnecessary antibiotic use for AOM.^6^ During watchful waiting, an antibiotic is started only if the child worsens or does not improve in 2-3 days. Watchful waiting may include (1) observation, where symptoms are monitored and an antibiotic is only prescribed if needed; or (2) use of a delayed prescription, where an antibiotic is prescribed with instructions to only fill and give to the child if the child worsens or does not improve. Randomized clinical trials have shown that watchful waiting can reduce antibiotic use for AOM by approximately 60% with similar patient outcomes and parent satisfaction to immediate antibiotic use.^7^ Despite these favorable data, evidence suggests watchful waiting is utilized in only 5%-20% of cases.^8-10^ The objectives of this study are to describe use of immediate antibiotics compared with watchful waiting in real-world clinical practice, identify factors associated with use of watchful waiting, and evaluate clinical outcomes associated with each strategy.

## METHODS

This multi-center, retrospective observational study examined children diagnosed with AOM in ambulatory practice settings across 3 health systems: (1) AllianceChicago (AC) in Chicago, IL, USA; (2) Denver Health (DH) in Denver, CO, USA; and (3) Intermountain Health (IH) based in Murray, UT, USA (**Supplementary Methods)**. Data were gathered from 27 AC, 36 DH, and 140 IH care locations.

Children aged 6 months-17 years with an index visit between July 1, 2018 and December 31, 2023 with any *International Classification of Diseases, Tenth Revision, Clinical Modification* (ICD-10) diagnosis code for AOM were eligible (**Supplementary Methods**).^11^ An index visit for AOM was defined as a visit with an ICD-10 diagnosis of AOM where there was *not* a visit with an ICD-10 diagnosis of AOM in the preceding 30 days. Those who were prescribed an antibiotic for any indication within 30 days of the index visit, had a history of tympanostomy tubes, or a concomitant diagnosis that may warrant an antibiotic at the index visit were excluded.

Index visits were categorized as having utilized (1) an immediate antibiotic, defined as an antibiotic prescribed within 2 days after the index visit that was not labeled as delayed, or (2) watchful waiting, defined as absence of an antibiotic prescribed within 2 days of the index visit or an antibiotic prescription at the index visit that was labeled as delayed. The capability to label an antibiotic prescription as delayed at the time of the encounter was only available at IH beginning in April 2019 (Figure S1). At DH and AC, there was not an established process for providers to identify an antibiotic prescription as delayed; validation of data by manual chart review did not reveal documentation of any instructions for prescriptions to be delayed; thus, they were considered negligible at these 2 sites.

To evaluate outcomes associated with each strategy, we captured all antibiotics prescribed between 3 and 30 days after the index visit. Prescription of a new antibiotic at a visit with an ICD-10 diagnosis of AOM within 3-14 days after the index visit was considered a treatment failure. Prescription of a new antibiotic at a visit with an ICD-10 diagnosis of AOM within 15-30 days after the index visit was considered recurrent AOM. Medically attended adverse events were captured by ICD-10 diagnoses as previously described (**Supplementary Methods**).

A randomly selected sample of 30 index visits per site was reviewed to determine whether there was a clinician-documented diagnosis of AOM, whether an antibiotic was prescribed at the index visit (and if so, whether it was an immediate or delayed prescription), and whether a new antibiotic was prescribed during the 30-day follow-up period. For variables where there was not at least 90% concordance between the manual and electronic data abstraction, a data analyst iteratively revised the data abstraction code until at least 90% concordance was achieved.

## RESULTS

A total of 140 579 index visits for AOM occurred in 100 431 unique children over the 5.5-year study period. An immediate antibiotic was prescribed at 118 613 (84.4%) of those visits while watchful waiting was utilized at 21 966 (15.6%) (Table 1). Of 117 181 cases in the immediate antibiotic group, 116 389 (99.3%) were prescribed the antibiotic on the day of the index visit. Of the watchful waiting cases, 12 356 (56%) were not prescribed an antibiotic (ie, initial observation) and 9610 (44%) were prescribed a delayed antibiotic.

**Table 1:** Demographic and Clinical Characteristics (by Unique Index Visit)

	**Total** ***n* = 140 579**	**Immediate antibiotic prescription** ***n* = 118 613**	**Observation or delayed antibiotic prescription** ***n* = 21 966**
Number of unique children	100 431	88 381	19 650
Age, months (median, range)	47 [6-216]	45.8 [6.0-216.3]	53.6 [6.0-216.2]
Sex, *n* (%) female	66 718 (47%)	56 284 (47%)	10 434 (48%)
Race, *n* (%)			
White, *n* (%)	119 268 (85%)	100 885 (85%)	18 383 (84%)
Black	4 682 (3%)	3 799 (3%)	883 (4%)
Asian	2 703 (2%)	2 245 (2%)	458 (2%)
American Indian or Alaska Native	1 075 (1%)	899 (1%)	175 (1%)
Multiple	2 218 (2%)	1 889 (2%)	329 (1%)
Native Hawaiian/other Pacific Islander	2 553 (2%)	2 132 (2%)	421 (2%)
Other	2 053 (1%)	1 878 (2%)	175 (1%)
Unknown/Declined	6 028 (4%)	4 886 (4%)	1 142 (5%)
Ethnicity, *n* (%)			
Hispanic/Latino	37 972 (27%)	31 748 (27%)	6 223 (28%)
Non-Hispanic/Latino	99 515 (71%)	84 217 (71%)	15 298 (70%)
Unknown/Declined	3 092 (2%)	2 647 (2%)	445 (2%)
Language preference^a^, *n* (%)			
English	128 053 (91%)	108 140 (91%)	19 913 (91%)
Spanish	11 265 (8%)	9 402 (8%)	1 863 (8%)
Other	1 241 (1%)	1 054 (1%)	187 (1%)
Visit location, *n* (%)			
Family practice	18 074 (13%)	15 929 (13%)	2 145 (10%)
Pediatrics	44 338 (32%)	37 404 (32%)	6 934 (32%)
Multi-specialty	334 (0.2%)	313 (0.3%)	21 (0.1%)
Internal medicine	20 (0.01%)	19 (0.02%)	1 (0.01%)
Urgent care	65 161 (46%)	55 729 (47%)	9 432 (43%)
School-based health clinics	730 (1%)	568 (0.5%)	162 (1%)
Missing/Unknown	11 922 (8%)	8 651 (7%)	3 271 (15%)
Insurance type, *n* (%)			
Commercial	88 009 (63%)	74 456 (63%)	13 553 (62%)
Government	46 218 (33%)	38 538 (32%)	7 680 (35%)
Uninsured	6 056 (4%)	5 341 (5%)	715 (3%)
Other	296 (0.2%)	278 (0.2%)	18 (0.1%)
ICD-10 codes, *n* (%)			
H66 (suppurative AOM)	124 191 (88%)	110 390 (93%)	13 801 (63%)
H65 (non-suppurative AOM)	15 945 (11%)	8 383 (7%)	7 562 (34%)
H67 (otitis media in diseases classified elsewhere	33 (0.02%)	24 (0.02%)	9 (0.04%)
H72 (perforation of TM)	1 738 (1%)	957 (1%)	781 (4%)
Concurrent conjunctivitis, *n* (%)	5 899 (4%)	5 272 (4%)	627 (3%)
Sick visits within 30 days prior to index visit, *n* (%)	54 583 (39%)	46 520 (39%)	8 063 (37%)
Hospitalizations within 1 year^b^, *n* (%)	2 215 (2%)	1 895 (2%)	320 (1%)

a Language was unknown for 20 children

b Count of hospitalizations excluded children under 12 months of age to avoid counting birth as a hospitalization

Between 3 and 30 days after the index visit (encompassing both treatment failure and recurrence), a new antibiotic was prescribed for any reason in 9850 (7%) cases and at a follow-up visit for AOM in 4102 (3%) (Table 2). A new antibiotic prescription at a visit for AOM between 3 and 14 days after the index visit (treatment failure) was low whether an immediate antibiotic or watchful waiting was utilized at the index visit (1% in each group). This finding was unchanged when the analysis was limited to index visits with a diagnosis of suppurative otitis media (Tables S2 and S3). Similarly, a new antibiotic prescription at a visit for AOM between 15 and 30 days after the index visit (recurrent AOM) was uncommon in both groups (2% and 1%, respectively). Medical visits for adverse events occurred in fewer than 1% of cases in both groups.

**Table 2:** Clinical Outcomes

	**Total index visits** ***n* = 140 579**	**Immediate antibiotic prescription** ***n* = 118 613**	**Observation or delayed antibiotic prescription** ***n* = 21 966**
Any antibiotic prescription between *days 3 and 30* after index visit	9 850 (7%)	8 501 (7%)	1 349 (6%)
Any antibiotic prescription between *days 3 and 14* after index visit	4 877 (3%)	4 074 (3%)	803 (4%)
Any antibiotic prescription between *days 15 and 30* after index visit	5 204 (4%)	4 629 (4%)	575 (3%)
Prescription for an antibiotic at a visit with a diagnosis code for AOM between *days 3 and 30* after index visit	4 012 (3%)	3 549 (3%)	463 (2%)
Prescription for an antibiotic at a visit with a diagnosis code for AOM between *days 3 and 14* after index visit	1 904 (1%)	1 638 (1%)	266 (1%)
Prescription for an antibiotic at a visit with a diagnosis code for AOM between *days 15 and 30* after index visit	2 130 (2%)	1 933 (2%)	197 (1%)
Medical diagnoses within 30 days after index visit			
Mastoiditis, *n* (%)	3 (0.002%)	3 (0.003%)	0 (0%)
Acute sinusitis, *n* (%)	253 (0.2%)	200 (0.2%)	53 (0.2%)
Pneumonia, *n* (%)	285 (0.2%)	231 (0.2%)	54 (0.2%)
Any medically attended adverse event, *n* (%)	923 (0.7%)	805 (0.7%)	118 (0.5%)

## DISCUSSION

In this large, multicenter, observational study of visits for AOM, watchful waiting was utilized in about 15% of cases while an immediate antibiotic was prescribed in about 85%. Subsequent healthcare encounters where a new antibiotic was prescribed were infrequent overall.

Although randomized trials have demonstrated similar clinical outcomes between immediate antibiotic prescriptions and watchful waiting, outcomes data outside of the idealized clinical trial setting are sparse. We demonstrated that new antibiotic prescriptions for any reason within 3-30 days after the index visit were low when watchful waiting was utilized (6%) and similar in frequency to the immediate antibiotic group. Prescriptions specifically at a visit for AOM were even less common (2%) and adverse events were rare. These data provide evidence that in a variety of clinical settings across different organizations, watchful waiting is successful in most cases. Watchful waiting was utilized more frequently in cases of nonsuppurative AOM suggesting this management strategy tends to be utilized in less severe infections. However, even when the analysis was limited to cases of suppurative otitis media, there was not a higher rate of new antibiotic prescriptions after the index visit with watchful waiting as compared with an immediate prescription.

This study has several strengths. Our dataset included diverse clinical locations across 3 large health systems, increasing the potential generalizability of the findings. In addition, patient-level data were used to identify diagnoses of AOM, associated antibiotic prescriptions, and clinical outcomes. These are real-world data of children diagnosed with AOM in clinical practice rather than those who have opted to participate in a clinical trial or who met stringent clinical criteria for AOM.

There are several limitations to this study. First, use of ICD-10 codes to identify cases invariably results in a degree of misclassification; however, we utilized a previously validated data extraction process^11^ and performed additional validation to ensure a high degree of data accuracy. Second, providers frequently misdiagnose AOM in the absence of tympanic membrane bulging.^12^ We were unable to verify the accuracy of diagnoses of AOM, and it is likely that some cases would not have met strict clinical criteria for AOM. Third, a subset of prescriptions classified as immediate may have been intended to be delayed. Although we did not find documentation of this during the validation process, instructions to delay filling a prescription may have been given verbally and not documented in the medical record. Fourth, we were unable to directly compare clinical outcomes between immediate antibiotic prescribing and watchful waiting in the absence of key diagnostic and clinical data regarding the severity of AOM episodes and because immediate antibiotic prescriptions were more common for children with suppurative AOM while watchful waiting was more common for children with non-suppurative AOM. Nevertheless, our findings demonstrate an overall low rate of failure of the watchful waiting strategy. Finally, the study population was largely White, English-speaking children with commercial insurance, which may limit generalizability.

In summary, watchful waiting was utilized in only about 15% of cases of clinician-diagnosed AOM across ambulatory care sites of 3 unique healthcare organizations. When watchful waiting was utilized, clinical outcomes were favorable as evidenced by the infrequent need for a new antibiotic prescription in the 30 days after the index visit. Interventions to scale up use of watchful waiting for AOM may therefore be a high-yield approach to reduce unnecessary antibiotic exposure for children with AOM.

The DISAPEAR Study Group members

Leisha Andersen, Aiden Gilbert, Aylin Hasan, Hannah Jensen, Amy Keith, Mirta Milanes, Theresa Morin, Sonja O’Leary, Barbora Nelson, Payal Patel, Deborah Rinehart, Allan M. Seibert, Valoree Stanfield, and Park Willis.

## Supplementary Material

JPIDS_Supplement_piaf104

## Data Availability

The study data are not available as it includes patient identifiers.
