# Supplementary material for: Watchful Waiting for Children With Acute Otitis Media: Frequency of Use and Outcomes in Clinical Practice
Source: J Pediatric Infect Dis Soc. 2025 Nov 12;14(12):piaf104. doi: 10.1093/jpids/piaf104 (PMC12678167; doi:10.1093/jpids/piaf104)
Supplement: JPIDS_Supplement_piaf104 [file jpids_supplement_piaf104.docx]

**Supplemental Materials**

**Watchful Waiting for Children with Acute Otitis Media: Frequency of Use and Outcomes in Clinical Practice**

**Section 1.** Supplemental Methods

AllianceChicago (AC) is a network of community health centers. Denver Health (DH) is an integrated, safety-net health care system. Intermountain Health (IH) is an integrated health delivery system which includes family medicine, pediatrics, and urgent care clinics.

Eligible International Classification of Diseases, Tenth Revision, Clinical Modification (ICD-10) codes included H65 (nonsuppurative otitis media), H66 (suppurative otitis media and unspecified otitis media), H67 (otitis media in diseases classified elsewhere), and H72 (perforation of tympanic membrane). For each index visit, care location, patient age, demographic characteristics, and subsequent clinical encounters and antibiotic(s) prescribed within 30 days of the index visit were extracted from the organization’s data warehouse.

Medically attended adverse events were defined as skin rash, allergic reactions, gastrointestinal symptoms, *Clostridioides difficile* infection, yeast infection, and acute kidney injury and were captured by ICD-10 code.^1,2^

Data were summarized descriptively with count and percentage for categorical variables. Continuous variables were analyzed with mean and standard deviation (SD) or median and interquartile range (IQR), as appropriate. Bivariate and multivariable logistic regression models were used to assess demographic and clinic characteristics associated with the use of the watchful waiting management strategy. Multivariable models were adjusted for age and suppurative AOM. Statistical analysis was assessed at the 0.05 level and all analysis was done using R version 4.2.1.

**Section 2.** Supplemental Results

Patient sex, race, ethnicity, preferred language, and insurance type were similar between visits where an immediate antibiotic or watchful waiting approach were utilized; however, children who received an immediate antibiotic tended to be younger (median age 45.8 months [IQR 20.8–82.7] vs 53.6 months [IQR 25.0–98.9], were more likely seen in an urgent care center, or have an ICD-10 diagnosis of suppurative AOM (H66).

Of the index visits where either an immediate or delayed antibiotic was prescribed, amoxicillin was the most commonly used agent (80% of prescriptions), followed by cefdinir (10%) and amoxicillin-clavulanate (7%) (**Supplemental Table 1**). Most antibiotic prescriptions were for 10- or 7-day durations (64% and 26% of prescriptions, respectively), while 5-day durations were uncommon (7% of prescriptions). Of the 21,966 visits where watchful waiting was utilized, a delayed antibiotic prescription was given in 9,586 (44%), though these delayed prescriptions were only identifiable in IH.

In the logistic regression model, numerous factors were associated with use of watchful waiting after adjusting for age and a diagnosis of suppurative AOM (**Supplemental Table 2**). Significant variables included age greater than or equal to two years, non-English preferred language, Hispanic ethnicity, Black race, urgent care or Pediatric clinic visit (versus Family Medicine clinic), government insurance, non-suppurative AOM, concurrent conjunctivitis, and a sick visit within the last 30 days. Although the reasons for this differential use of watchful waiting as compared with immediate antibiotics in these groups could not be elucidated from this study, this may be an important area for future study.

With the exception of IH, we lacked the ability to definitively determine whether a given antibiotic prescription was intended to be immediate or delayed. Although our validation demonstrated that all antibiotic prescriptions at the index visit appeared to be intended to be immediately filled and taken (i.e., no documentation of instructions to delay filling the prescription), this may have led to an underestimation in the proportion of cases in which watchful waiting via delayed prescriptions was utilized. However, this effect is likely small and would not change the overall conclusions of the study.

A small proportion of patients (<1%) were prescribed an antibiotic one or two days after the index visit. Although these patients were classified as having received an immediate antibiotic, some may have represented failure of observation. Since the overall number of these cases was small, this would have been unlikely to influence the overall conclusions.

**Supplemental Table 1.** Antibiotic treatment

|  | **Total**  **N = 140,579** | **Immediate antibiotic prescription**  **N = 118,613** | **Observation or delayed antibiotic prescription**  **N = 21,966** |
| --- | --- | --- | --- |
| Any antibiotic prescribed, n (% of index visits) | 128,199 (91%) | 118,613 (100%) | 9,586 (44%) |
| Specific antibiotics prescribed, n (% of index visits with an antibiotic) (n = 128,199) | |  |  |
| Amoxicillin | 102,351 (80%) | 93,968 (79%) | 8,383 (87%) |
| Amoxicillin/clavulanate | 9,253 (7%) | 8,801 (7%) | 452 (5%) |
| Cefdinir | 13, 197 (10%) | 12,494 (11%) | 703 (7%) |
| Azithromycin | 1,044 (1%) | 1,039 (1%) | 5 (0.1%) |
| Other | 2,354 (2%) | 2,311 (2%) | 43 (0.5%) |
| Duration of therapy (all antibiotics, N = 121,423) | |  |  |
| Median [IQR] | 10 [7 – 10] | 10 [7 – 10] | 10 [7 – 10] |
| 5 days, n (%) | 8,601 (7%) | 8,173 (7%) | 428 (4%) |
| 7 days, n (%) | 31,200 (26%) | 27,740 (23%) | 3,460 (36%) |
| 10 days, n (%) | 77,945 (64%) | 72,385 (61%) | 5,560 (58%) |
| 14 days, n (%) | 726 (0.6%) | 719 (0.6%) | 7 (0.1%) |
| Other duration^b^ | 3,384 (3%) | 3,251 (3%) | 133 (1%) |

^a^6,776 participants received an antibiotic but were missing a sig/duration value.

^b^Other duration of antibiotics included 1-4, 6, 6-13, 15, 16, 18, 20, 21, 30.

**Supplemental Table 2.** Logistic regression model of factors associated with use of watchful waiting in clinical practice. Adjusted analysis included age groups and suppurative AOM (by ICD-10 diagnosis code).

|  | **Variable** | **Bivariate analysis**  Odds ratio (95%CI) | ***P*** | **Adjusted analysis**  **Odds ratio (95%CI)** | ***P*** |
| --- | --- | --- | --- | --- | --- |
| Age | |  |  |  |  |
|  | < 2 years | Reference |  | Reference |  |
|  | >= 2 years | 1.33 (1.28, 1.37) | <.0001 | 1.24 (1.19, 1.28) | <.0001 |
| Preferred language | |  |  |  |  |
|  | English | Reference |  | Reference |  |
|  | Non-English | 1.06 (1.01, 1.12) | 0.0136 | 1.09 (1.03, 1.15) | 0.0042 |
| Location of visit | |  |  |  |  |
|  | Urgent Care | 1.26 (1.20, 1.32) | <.0001 | 1.51 (1.43, 1.59) | <.0001 |
|  | Pediatrics | 1.39 (1.32, 1.46) | <.0001 | 1.67 (1.58, 1.76) | <.0001 |
|  | Family Medicine | Reference |  | Reference |  |
| Insurance type | |  |  |  |  |
|  | Commercial | Reference |  | Reference |  |
|  | Government/Self-Pay/Other | 1.05 (1.02, 1.08) | 0.0026 | 1.11 (1.07, 1.14) | <.0001 |
| Non-suppurative vs suppurative (as coded by ICD-10) | |  |  |  |  |
|  | Non-suppurative (H65) | 7.24 (6.99, 7.51) | <.0001 | 7.42 (7.16, 7.70) | <.0001 |
|  | Suppurative (H66) | Reference |  | Reference |  |
| Concurrent conjunctivitis | | 0.63 (0.58, 0.69) | <.0001 | 0.66 (0.61, 0.72) | <.0001 |
| Sick Visits within 30 days Prior | | 0.85 (0.82, 0.87) | <.0001 | 0.76 (0.73, 0.78) | <.0001 |
| Race | |  |  |  |  |
|  | Asian | 1.21 (1.09, 1.33) | 0.0004 | 1.12 (0.99, 1.25) | 0.0572 |
|  | Black or African American | 1.84 (1.70, 1.99) | <.0001 | 1.48 (1.35, 1.61) | <.0001 |
|  | Other | 0.86 (0.80, 0.91) | <.0001 | 0.76 (0.71, 0.82) | <.0001 |
|  | White | Reference |  | Reference |  |
| Ethnicity | |  |  |  |  |
|  | Hispanic or Latino | 1.08 (1.04, 1.11) | <.0001 | 1.15 (1.11, 1.19) | <.0001 |
|  | Not Hispanic or Latino | Reference |  | Reference |  |

**Supplemental Table 3.** Clinical outcomes for subgroup of children with suppurative AOM

|  | **Total index visits**  **N = 124,191** | **Immediate antibiotic prescription**  **N = 110,390** | **Observation or delayed antibiotic prescription**  **N = 13,801** |
| --- | --- | --- | --- |
| Any antibiotic prescribed at index visit (day 0-2), n (% of index visits) | 119,042 (96%) | 110,390 (100%) | 8,562 (62%) |
| Any antibiotic prescription between day 3 and 30 after index visit | 8,857 (7%) | 7,975 (7%) | 882 (6%) |
| Any antibiotic prescription between day 3 and 14 after index visit | 4,333 (3%) | 3,815 (3%) | 518 (4%) |
| Any antibiotic prescription between day 15 and 30 after index visit | 4,728 (4%) | 4,350 (4%) | 378 (3%) |
| Prescription for an antibiotic at a visit with a diagnosis code for AOM between day 3 and 30 after index visit | 3,580 (3%) | 3,296 (3%) | 284 (2%) |
| Prescription for an antibiotic at a visit with a diagnosis code for AOM between day 3 and 14 after index visit | 1,681 (1%) | 1,522 (1%) | 159 (1%) |
| Prescription for an antibiotic at a visit with a diagnosis code for AOM between day 15 and 30 after index visit | 1,919 (2%) | 1,794 (2%) | 125 (1%) |

**Supplemental Table 4.** Medically attended adverse events

|  | **Total index visits**  **N = 140,579** | **Immediate antibiotic prescription**  **N = 118,613** | **Observation or delayed antibiotic prescription**  **N = 21,966** |
| --- | --- | --- | --- |
| Dermatologic (rash, SJS, TEN, urticaria), n (%) |  |  |  |
| Skin rash, n (%) | 236 (0.2%) | 212 (0.2%) | 24 (0.1%) |
| SJS or TEN | 0 (0%) | 0 (0%) | 0 (0%) |
| Urticaria | 85 (0.06%) | 75 (0.06%) | 10 (0.05%) |
| Gastrointestinal |  |  |  |
| Abdominal pain | 56 (0.04%) | 48 (0.04%) | 8 (0.04%) |
| Nausea or vomiting | 145 (0.1%) | 125 (0.1%) | 20 (0.09%) |
| Non-*C. difficile* diarrhea | 87 (0.06%) | 64 (0.05%) | 23 (0.1%) |
| Secondary infection |  |  |  |
| *C. difficile* infection | 3 (0.002%) | 3 (0.003%) | 0 (0%) |
| Yeast infection | 377 (0.3%) | 337 (0.3%) | 40 (0.2%) |
| Acute kidney injury | 2 (0.001%) | 1 (0.001%) | 1 (0.001%) |

**Supplemental Figure**. Electronic health record order for a delayed antibiotic prescription at Intermountain Health (available beginning in April 2019)


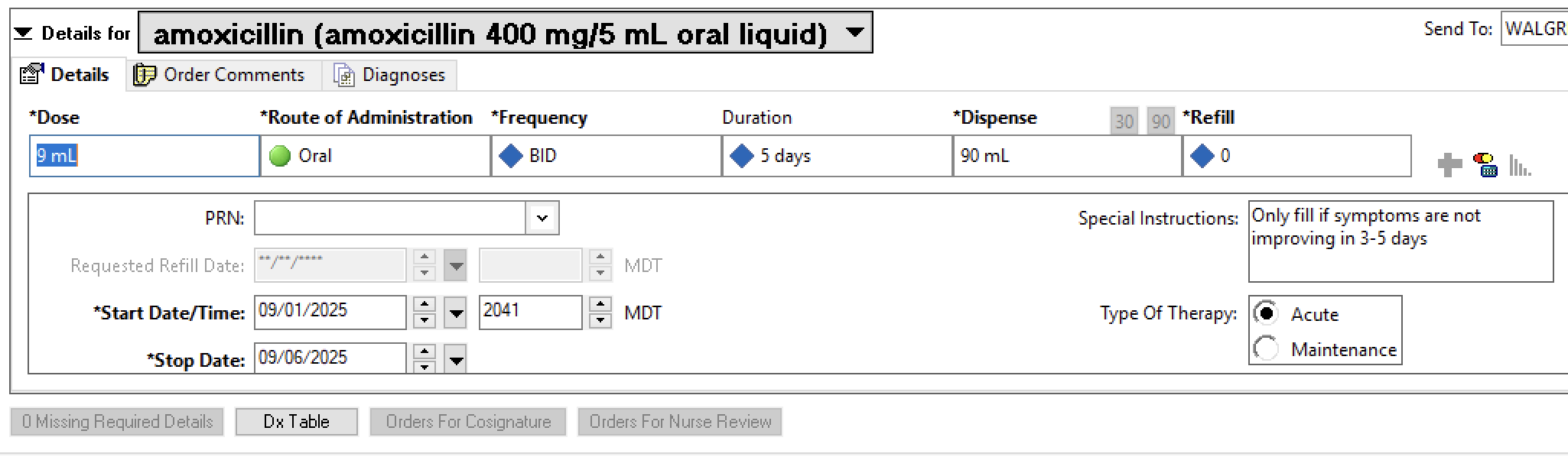


**References**

1. Butler AM, Brown DS, Durkin MJ, et al. Association of Inappropriate Outpatient Pediatric Antibiotic Prescriptions With Adverse Drug Events and Health Care Expenditures. *JAMA Netw Open*. 2022;5(5):e2214153. doi:10.1001/jamanetworkopen.2022.14153

2. Savage TJ, Kronman MP, Sreedhara SK, Lee SB, Oduol T, Huybrechts KF. Treatment Failure and Adverse Events After Amoxicillin-Clavulanate vs Amoxicillin for Pediatric Acute Sinusitis. *JAMA*. 2023;330(11):1064-1073. doi:10.1001/jama.2023.15503
